# Nanopore sequencing of non-oncogenic oral Papillomaviruses from people living with HIV

**DOI:** 10.21203/rs.3.rs-6082806/v1

**Published:** 2025-03-07

**Authors:** Ian G Munabi, Kamulegeya Adrian, Muwuluza Mark, Nalwanga Sylvia1, David P Kateete, Fred C Semitala, Erisa Mwaka, Jennifer E Cameron, William Buwembo

**Affiliations:** Department of Anatomy, School of Biomedical Sciences, Makerere University College of Health Sciences, Kampala, Uganda; Department of Dentistry, School of Dentistry, Makerere University College of Health Sciences, Kampala, Uganda; Department of Anatomy, School of Biomedical Sciences, Makerere University College of Health Sciences, Kampala, Uganda; Department of Anatomy, School of Biomedical Sciences, Makerere University College of Health Sciences, Kampala, Uganda; Department of Immunology & Molecular Biology, School of Biomedical Sciences, Makerere University College of Health Sciences, Kampala, Uganda; Department of Medicine, School of Medicine, Makerere University College of Health Sciences, Kampala, Uganda; Department of Anatomy, School of Biomedical Sciences, Makerere University College of Health Sciences, Kampala, Uganda; Louisiana State University Health Sciences Center: New Orleans, LA, USA; Department of Anatomy, School of Biomedical Sciences, Makerere University College of Health Sciences, Kampala, Uganda

**Keywords:** Nanopore, sequencing, Non-oncogenic, Oral Papillomaviruses, Diversity, People living with HIV

## Abstract

**Objective:**

To explore the diversity of non-oncogenic papillomaviruses in saliva samples from people living with HIV using nanopore amplicon-based sequencing for detection and typing.

**Methods:**

This was a secondary analysis of data from the nanopore sequencing of amplicons obtained from polymerase chain reaction detection of papillomaviruses from 127 samples of people living with HIV. The sequencing data was cleaned and analyzed using a series of bash, Python and R scripts to produce output based on comparisons with the PAVE reference database for all known non-oncogenic papillomaviruses.

**Results:**

A total of 171,194 reads corresponding to 201 known papillomavirus types were obtained from the data. Most of these reads (69%), belonged to the human non-oncogenic papillomavirus types. The most abundant nonhuman and non-oncogenic PV, Trichechus manatus latirostris papillomavirus 4 in 99% of the samples. There were nine other less abundant non-oncogenic papillomaviruses that were found in 95% or more of the samples as mixed infections.

**Conclusions:**

This study demonstrates that there are many non-oncogenic PV infections in samples from PLHIV, most of which are mixed infections from this setting. It is important to note that the non-human non-oncogenic PVs, as a potential one health concern, were highly prevalent in this population.

## Introduction

The Papillomaviruses (PV) group of viruses have overtime become established as a major contributor to human oropharyngeal and oral cancers (Moore & Mehta, 2015). In most cases these PV infections result in self limiting infections that are typically cleared by the hosts immunity for most healthy people (Wood, Bain, Smith, Whiteman, & Antonsson, 2017). Even then there are cases where the infections may persist, which is associated with the development of cancer (Moran-Torres et al., 2021; Tam et al., 2018). As sexually transmitted infections, young people are at an increased risk of infection by PV due to their higher sexual activity that may be associated with quick reinfection (Moran-Torres et al., 2021). Human sexual activity may also introduce oncogenic genital PV types into the oral cavity (Moran-Torres et al., 2021; Morhason-Bello et al., 2021). The oncogenic PV types have achieved this status due to their propensity to induce oncogenesis. The twelve PV types currently classified as high-risk, based on their potential to induce malignancy include the PV types 16, 18, 31, 33, 35, 39, 45, 51, 52, 56, 58 and 59 (Chung, Bagheri, & D’Souza, 2014; Petca et al., 2020). Human PV type 16 (HPV16) infection remains one of the most important human carcinogens, causing more than 300,000 deaths per year worldwide (Bouvard et al., 2009). The oropharynx is the subsite within the head and neck region that is strongly associated with PV infection (Kreimer et al., 2005), and that among PV-associated oropharyngeal cases, the overwhelming majority (~90%) are due to PV 16 (Chaturvedi et al., 2013; Chung et al., 2014; D’Souza et al., 2007). It has been noted that the overall prevalence of PV using P16 marker in Ugandan Oral squamous cell carcinoma (OSCC) sampled was 20.3 (95% CI 10.9 to 32.8) (Kabagenyi, Otiti, Namwagala, Kamulegeya, & Kalungi, 2020). In the USA, African Americans with p16 positive OSCC had worse clinical outcomes compared with p16 positive European Americans (O’Neill et al., 2021). Vaccines have been developed targeting a few of these known oncogenic PVs at a population level (Kumakech et al., 2016).

The non-oncogenic PV include all the other low risk human PVs and the PVs from other animals (Wolf et al., 2024). Low risk PV infections have been found in people with oral pharyngeal squamous cell carcinoma and tonsilitis (Strzelczyk et al., 2021). They are also associated with sexual activity or contact with infected materials (Petca et al., 2020; Wolf et al., 2024). In low resource settings the non-human PVs picked from other animals like cattle, dogs, and cats are important sources of a potentially positive PV test result. This is important for people living with HIV (PLHIV), who are more prone to having PV infections. This is because PLHIV have increased exposure of the basement membrane in inflammatory conditions like periodontitis (Groenewegen et al., 2019; Shipilova, Dayakar, & Gupta, 2017; Syrjanen, 2018), that may lead to increased and or longer duration of PV infection or exposure. Some of the other risk factors for this group with respect to oral PV infection include oral sexual contact (Beachler et al., 2015), age (Beachler et al., 2015), smoking (Alli et al., 2020; Beachler et al., 2015; Louvanto, Rintala, Syrjanen, Grenman, & Syrjanen, 2010), tonsillectomy, years after HIV diagnosis (Ablanedo-Terrazas et al., 2018), low CD4+ cell count (Muller et al., 2015; Riddell et al., 2022), and in women concurrent oral and genital PV infections (Tahmasebi et al., 2023). Also, PLHIV are more likely to present with mixed PV infections mainly due to the non-oncogenic PV types, compared to HIV negative individuals (Camargo et al., 2018). In HIV negative people, infection with multiple PV types, significantly increases the HIV risk acquisition by 20% for each additional PV type detected (Liu et al., 2022). Mixed PV infections may persist longer than non-mixed HPV infections (Louvanto et al., 2013; Louvanto et al., 2010). There are recommendations that the finding of a mixed PV infection is an indication for closer and frequent follow up and or intervention (J. Kim, Kim, & Park, 2022; M. Kim, Park, Jeong, & Park, 2021; Wittenborn et al., 2022). Thus, while vaccination may eventually wipe out most of the targeted high risk PV types, the non targeted PV types may become relatively more abundant, each of which has the capacity to initiate oncogenesis (Gage, Meyers, & Wettstein, 1990; Pimenoff et al., 2023).

In the low resource settings of Africa low-cost Polymerase Chain Reaction (PCR) degenerate primer-based gel detection and typing methods are still in use (Adebayo, Owotade, Folarin, Oninla, & Oyetola, 2024; Tadlaoui et al., 2024). Some of these methods rely on the use of various primer systems like the FAP 59 and FAP 64 degenerate base containing primer system (Sias et al., 2019), the double nested PGmy,9/11 and Gp+5/+6 primers (Fuessel Haws et al., 2004), and the Soltar method for the identification of a select number of PV types with emphasis on the oncogenic PV types (Sotlar et al., 2004). It is important to note that there are many other methods for PV detection using commercial kits and assays each with differing accuracy to catch all or specific PVs (Arroyo Muhr et al., 2022; Torres et al., 2023). The amplicon based next generation sequencing protocols of especially with the nanopore platform bring additional opportunities for extending the scope of the above PCR and gel detection methods (Chan et al., 2020; Timsit, Armand-Lefevre, Le Goff, & Salmona, 2024). This is important in view of the falling costs of the different PV detection methods where there are many samples for testing. This was one of the initial recognised limitations of sequencing whose costs have significantly reduced (Timsit et al., 2024). Even with the significant reduction in costs there is still a need for well documented methods or protocols with all the necessary details to aid replication (Arroyo Muhr et al., 2022). This is especially important for saliva samples that may not have as high a concentration of PV as other tissues. In this manuscript we set out to explore the diversity of non-oncogenic PVs in saliva samples from PLHIV using nanopore amplicon-based sequencing for detection and typing.

## Methods

Study setting and samples: This was a secondary analysis of sequencing data from a series of PV related lab studies on 130 samples at the Department of Human Anatomy on samples whose collection has been described elsewhere (William et al., 2024). Briefly the 130 samples were obtained from people living with HIV (PLHIV) from Mulago ISS clinic of Makerere University Joint AIDS Program (MJAP), situated in Kampala Uganda which serves a total of 18,000 patients drawn from both the urban and peri-urban populations of south-central part of Uganda in East Africa (Muddu et al., 2021). The samples used for this report were selected from the previously confirmed PV positive samples of the parent study that examined the associations between PV, microbiota and cancer in PLHIV (William et al., 2024). Only those reads with barcodes for linking to the sample data were included in the analysis. Also, during analysis any read belonging to one of the known oncogenic PVs or that was found to contain human DNA (integration) was excluded.

### DNA Extraction and amplification:

The pre identified known PV positive saliva pellet samples were taken out of the minus 80 degrees long term storage for DNA extraction using Quick-DNA Mini prep Plus Kit (D4069, Zymoresearch, CA, USA) following the manufactures instructions including overnight protein kinase digestion. After extraction the eluted DNA samples were quantified using a Nanodrop colorimeter (Thermofisher scientific, MA, USA) following the manufacturer’s instructions. Ten-micro-liters for each sample was used for amplification with the forward FAP 59 (5’TAACWGTIGGICAYCCWTATT3’), reverse FAP 64 (5’CCWATATCWVHCATITCICCATC3’) primers (Sias et al., 2019) and DreamTaq DNA polymerase in PCR reactions. The following assay conditions: 5 min at 94 °C, 40 cycles (denaturation 94 °C for 30 seconds, annealing 52 °C for 45 seconds, and extension 72 °C 1 min) followed by a final extension step at 72 °C for 7 min were used during PCR amplifications. The 490bp band PV positive PCR products were visualized using 2% agarose gel stained with 1ug/ml ethidium bromide. Samples without any band from the FAP primers were subjected to a confirmatory PCR screening for 150bp bands using the double nested PGmy9/11 and Gp+5/+6 primers and protocol (Fuessel Haws et al., 2004).

### Nanopore sequencing kits:

Sequencing for the 130 samples was carried out using two protocols based on the SQK-LSK114-XL sequencing kit and the native barcoding SQK-NBD114–96 kit, according to the manufacturer’s instructions for each. In the case of the SQK-LSK114-XL sequencing kit, the protocol for nanopore tagged amplicons(Kiryowa et al., 2024) was used with the following modifications. The PCR products from all the above FAP and or GP5+ runs, were cleaned up with Clean NGS beads (Coenecoop 75, 2741 PH Waddinxveen, Netherlands) with the ratio of 1:2 of the amplicons to the beads as per the manufacturer’s instructions for washing twice with 800μl of freshly prepared 70% ethanol. After washing the DNA was eluted in 10ul of Nuclease free water and incubated at room temperature for 10 min. The cleaned eluted amplicons were quantified using a Qubit^™^ 4 Fluorometer (Themo Fisher Scientific, City, Singapore). The purified PCR products were then tagged with the Nanopore EXP-PBC096 PCR barcoding expansion pack (Oxford Nanopore Technologies plc, Oxford, UK) under the following conditions 90-degrees Celsius for 2min 90°C for 30seconds,60°C for 30 seconds 68°C for 1min following 20 cycles and final extensionat 68°C for 5min. fo. The barcoded PCR products were purified with Clean Next Generation Sequencing (NGS) beads in a ratio of 1 to 0.4 for the sample to beads before pooling in equimolar volumes for Ligation using the SQK-LSK114-XL kit (Oxford Nanopore Technologies plc, Oxford, UK) as per the manufacturer’s instructions. The final DNA library was quantified using both Nano drop One (Themo Scientific, USA) and Qubit^™^ 4 Fluorometer (Themo Fisher Scientific, City, Singapore). The quantified DNA library not exceeding 10-fetomolar based on the Qubit^™^ 4 Fluorometer (Themo Fisher Scientific, City, Singapore) measurement for each run, was loaded onto the MinION MIN-101B device running a flongle Flow Cell FLO-FLG114 (Oxford Nanopore Technologies plc, Oxford, UK) until end-of-life setting. For the native barcoding SQK-NBD114–96 kit (Oxford Nanopore Technologies plc, Oxford, UK), non tagged primers were used, following the steps outlined in the protocol for the flongle device platform (version number NBE_9171_v114_revP_15sep2022) on eight (8) samples. The other major difference between the two kits is the need to repair the ends of all the reads before the addition of the barcodes using a set of end repair reagents from New England Biolabs (NEB).

### Processing of sequencing data:

The sequencing data from 130 samples was initially saved as POD5 files during sequencing. Using a python 3.12 Jupyter notebook and bash commands the POD5 files were converted first to bam files using the GPU version of the manufacturer’s demultiplexing software, Dorado version 8.1 running on a Nvidia GPU 3070, AUSUS Tuf Dash F15 i7 64GB ddr5 ram laptop. The highest quality ONT dorado v5 SUP models were to ensure that the reads obtained are of the best quality. Sequencing reads with a minimum quality Phred score of 5, were retained in bam files for subsequent analysis. This was followed by conversion of the reads to the fasta format using SAMtools (Danecek et al., 2021), and later to blast output summary text files of the aligned reads using the L1 reference sequences for all known non-oncogenic PVs from Pave database (www.pave.niaid.nih.gov [Date of download: 12 January 2025]), for importing into R. During the blast step, only reads longer than 100 bases after trimming the nanopore sequencing adapters, primers and tags, were retained for the blasting against the reference genomes, using the highest settings of the blastn based NanoBLASTer (Amin, Skiena, & Schatz, 2016). Downstream analysis in the R statistical computing environment made use of the data.table related R packages for both the initial data wrangling and later generation of summaries from the data using the identified PV related reads from both kits.

### Analysis of the data:

#### Data analysis:

The data was imported as excel sheets or text files (TSV) into the R version 4.4.2 statistical computing environment running on windows for data wrangling to merge and label all the relevant pieces of data before the production of summaries. Descriptive summaries of the data from the two kits were made using frequencies. Additional, multilevel regression analyses using the glmm TMB, with a Poisson distribution to compute the rate ratios of the PV infection count outcomes controlling for the participant related variables. This was followed by the reorganization of the data into a phyloseq object using in house scripts. That was analysed to record the total number of PV OTUs followed by a series of comparisons using the Phyloseq and Vegan packages in R to generate the alpha and beta diversity indices comparing the PV OTUs and number of reads for each PV with respect to gender, and age for the human compared to the non-human non-oncogenic PV types. A sub analysis was made to compare the OTU in the samples from the same individuals processed using different nanopore sequencing kits.

##### Ethical considerations

This work and the collection of samples used in this study has been approved by Ethics Committee of Makerere University School of Medicine Research and Ethics committee SOMREC (REC REF 2022-451) and the Uganda National Council of Science and technology UNCST (HS2541ES. The ethics approval date: November 22, 2022. All procedures followed were in accordance with the ethical standards of the committee responsible on human experimentation (institutional and national) and with the Helsinki Declaration. Informed consent was obtained from all participants, in writing and dated, before enrolment in the study. Additional consenting for sample storage and genomic analysis was done.

## Results

Table 1 provides a summary of the descriptive characteristics of the 127 research participants whose PV sequencing data is summarised in this document. In this table 1, note that on average the selected participants were 47 years of age, and they were mostly (67%) female. The nanopore sequencing data contained 2,791,802 reads of which 382,232 (13.69%) mapped onto the L1 gene of one of the known PV genomes in the reference database. There were 200,275 of the 382,232 (52.40%) PV reads found with a barcode corresponding to one of the participants’ sample data. There were 3,813 reads that mapped onto one of the 12 known oncogenic PVs and another 25,268 reads that were found to contain human DNA, that were removed. The final working dataset had a total of 171,194 non-oncogenic PV reads. There were 117,402 (68.58%) reads that mapped onto one of the known human PVs. The risk of having a human compared to a non-human non-oncogenic PV was not affected by increasing age (Rate ratio (RR) = 0.24 pvalue = 1), gender (RR < 0.01, pvalue = 1), level of education (RR< 0.01, pvalue = 1), participants religious faith or denomination (RR< 0.01, pvalue = 1), and the participants occupation (RR< 0.01, pvalue = 1).

A total of 206 unique OTUs were identified, based on the L1 regions of the known PV genomes in the reference database. Table 2, which provides a summary of both the alpha diversity indices by the different participant variables of gender, religion, level of education and occupation. Note the similar values against the different diversity indices for each of the participants variables. For example, the male participants had an additional genome identified compared to the female participants. Increasing age (F=0.36, pvalue = 0.57), gender (F= 4.11, pvalue = 0.06), level of education (F = 0.42, pvalue = 0.56), participants religious faith or denomination (F = 2.01, pvalue = 0.17), and the participants occupation (F = 0.50, pvalue = 0.48) did not create significant differences in the types of PV identified. Table 3 provides a summary of the non-oncogenic PV types with more than 1,000 reads as observed in this population. The most common nonhuman and non-oncogenic PV, Trichechus manatus latirostris papillomavirus 4 (TmPV4), was found in the data from most of the sequenced samples. This is seen in the last column of table 3, where 126/127 (99.21%) of the samples had TmPV4. The other PVs affecting 95% or more of the participants were HPV111 (125/127, 98.43%), HPV173 (125/127, 98.43%), PphPV1 (125/127, 98.43%), HPV162 (124/127, 97.64%), CmPV1 (124/127, 97.64%), HPV170 (123/127, 96.85%), HPV138 (123/127, 96.85%), HPV137 (122/127, 96.06%), and HPV123 (121/127, 95.28%).

### Comparison of the kits used

Four samples were run on both the nanopore expansion barcoding kit and the native barcoding kit each with two membranes. There was a total of 1,505 reads from the four samples run on the membranes where the expansion barcoding kit was used. In comparison there was a total of 62,693 reads from the two membranes on which the native barcoding kit was. Table 3 provides a summary of the total number of PV identified reads from each membrane for the four samples. Table 4 also provides a summary of the alpha diversity indices comparisons for each kit used. In this table kindly note, the observed OTUs were more with the native barcoding kit (Number = 199) compared to the expansion barcoding kit (Number = 90). Also in table 3, all the other diversity indices were higher for the native barcoding kit. Despite this, the beta diversity scores comparing the two kits were both very close to 1 (Bray Curtis = 0.97 and Jaccard = 0.99), suggesting a very high level of similarity.

## Discussion

We set out to explore the diversity of non-oncogenic PVs in saliva samples from PLHIV using nanopore amplicon-based sequencing for detection and typing. We found both human and non-human non oncogenic PVs in all the saliva samples whose DNA was sequenced. As shown in Table 3, five of the identified non-human non-oncogenic PVs were from cats (FcaPV2), rats (RnPV2), reptiles (CmPV1), cattle or goats (PphPV1), and dogs (CPV26), that usually share living spaces with humans in our low resource settings. This is supported by the observation that more than 90% of the samples had one or more of these non-human PVs. This and the observation in Table 1, that most of the participants were of both low levels of education and not in formal employment increases the chances of them living in spaces with one or more of the above combinations of animals. Infection could be the result of close contact with the animals or contamination of surfaces as opposed to sexual contact (Petca et al., 2020; Wolf et al., 2024). This raises three points of concern, the first of which is a one health concern associated with the potential risk of developing severe forms of disease when viruses that have adapted to a particular type of species cross the species barrier as has been described else where (Okamoto et al., 2015). This concern is even more important in people living with HIV who due to their compromised immunity may not be able to clear or mount an immune response to these non-human PVs as quickly health individuals. As has already been mentioned these low-risk PV infections have been associated with oral pharyngeal squamous cell carcinoma and tonsilitis (Strzelczyk et al., 2021). The above delayed clearance in PLHIV may contribute to their being more prone to having PV infections that in combination with increased exposure of the basement membrane in inflammatory conditions like periodontitis (Groenewegen et al., 2019; Shipilova et al., 2017; Syrjanen, 2018), that may lead to increased and or longer duration of PV infection or exposure. This one health concern is even more important in view of the observation that there was no difference in the infections compared to the purely human PVs. This may imply that there is constant exposure of the humans to the animal PVs leading to the observed infections across the species barrier. The increasing interest in one health aspects of disease transmission due to the recent pandemics points to a need for more specific testing in this population.

The second concern related to the finding of both the non-human PVs picked from other animals like cattle, dogs, and cats with the human non-oncogenic PVs in this population is that these PVs are an important source of a potentially positive PV test result. This is especially true where the PV test being used is based on some of the previously mentioned low-cost universal degenerate primer sets (Adebayo et al., 2024; Fuessel Haws et al., 2004; Sias et al., 2019; Tadlaoui et al., 2024). As has been noted else where (Daudt et al., 2016), concerns have been raised about missing PVs due to use of non-specific testing methods. This is important for the ongoing vaccination efforts that may eventually wipe out most of the targeted high risk PV types, leaving the non targeted PV types (Gage et al., 1990; Pimenoff et al., 2023). Thus, finding a positive PV test result in previously vaccinated persons, should not lead to the conclusion that vaccination has failed, rather it should be an indicator for increased efforts to identify which one of the other PVs is prevalent. For PLHIV this additional investigation is important due to the risk of developing cancer from one of these other PVs even though they are knowns as low risk or non-oncogenic PVs. The falling cost of next generation sequencing should make it possible to effectively identify these PVs even in low resource settings (Chan et al., 2020).

The third concern is related to finding multiple PV infections due to these non-oncogenic HPVs in almost every sample that was sequenced. In this study we found that TmPV4 (99.21%), HPV111 (98.43%), HPV173 (98.43%), PphPV1 (98.43%), HPV162 (97.64%), CmPV1 (97.64%), HPV170 (96.85%), HPV138 (96.85%), HPV137 (96.06%), and HPV123 (95.28%) were in more than 95% of the samples. This means that in this sample each of the participants had between 7 to 10 PV types at any one instant. It also confirms the previous mentioned observation of PLHIV being more likely to present with mixed PV infections mainly with non-oncogenic PV types (Camargo et al., 2018). From literature these mixed infections are also associated with longer persistence which is associated with oncogenesis (Louvanto et al., 2013; Louvanto et al., 2010). Mixed infections are also associated with increased risk of HIV transmission. Thus, the recommendation that on the finding of a mixed PV infection is an indication for closer and frequent follow up and or intervention (J. Kim et al., 2022; M. Kim et al., 2021; Wittenborn et al., 2022).

In this study we looked at the data from using two of the nanopore next generation sequencing kits on the same PV positive samples. The key difference in the two kits is the use of the additional DNA end repair kits in the case of the native barcoding kit. These additional repair kits made this protocol more expensive even though it identified 100 more PVs from the same set of samples. Overall, there was high similarity of PVs identified in terms of comparisons of the PV related sequencing data obtained from using the two kits. This is important because the nanopore platform is being promoted as a low-cost alternative for next generation sequencing studies in low resource settings (Chan et al., 2020). The choice of which kit to use should ideally be informed by the research questions being asked in relation to sequencing depth to catch the rare PV types or focused on the most abundant PVs when using amplicon-based protocols. There is need for further exploration of the opportunities presented by this platform for doing PV identification in low resource settings.

Some of the key limitations of our approach was the use of the reference data from the PAVE database (www.pave.niaid.nih.gov [Date of download: 12 January 2025]), that limited us to only those PVs that are known. This was further limited by the efficiency of the primers used to make the amplicons based on PVs for which they were designed (Adebayo et al., 2024; Fuessel Haws et al., 2004; Sias et al., 2019; Tadlaoui et al., 2024). There is a possibility that we may have missed one of the non-oncogenic PVs that though highly prevalent in this setting is not detectable with the above current methods. This possibility has been noted else where (Daudt et al., 2016), and we declare it as a key limitation for the study. Despite this limitation we were able to demonstrate that there is a wide diversity of non-oncogenic PV infections in samples from PLHIV. We noted that most participants had more than one PV pointing to mixed infections and that the non-human non-oncogenic PVs were highly prevalent in this population of PLHIV.

## Data Availability

Data is provided with in the manuscript

## Abbreviations

PV: Papillomaviridae
PCR: Polymerase Chain Reaction
OSCC: Oral squamous cell carcinoma
USA: United States of America
PLHIV: people living with HIV
OTU: Organismal Taxonomic Unit
GPU: Graphics Processing Unit
TSV: Tab separated values
SOMREC: School of Medicine Research and Ethics committee
UNCST: Uganda National Council of Science and technology
